# Some Irregularities in the Evaluation of Surface Parameters of Solid Materials by Inverse Gas Chromatography

**DOI:** 10.1021/acs.langmuir.3c01649

**Published:** 2023-11-21

**Authors:** Tayssir Hamieh

**Affiliations:** Faculty of Science and Engineering, Maastricht University, P.O. Box 616, Maastricht 6200 MD, The Netherlands; Laboratory of Materials, Catalysis, Environment and Analytical Methods Laboratory (MCEMA), Faculty of Sciences, Lebanese University, Hadath, Lebanon

## Abstract

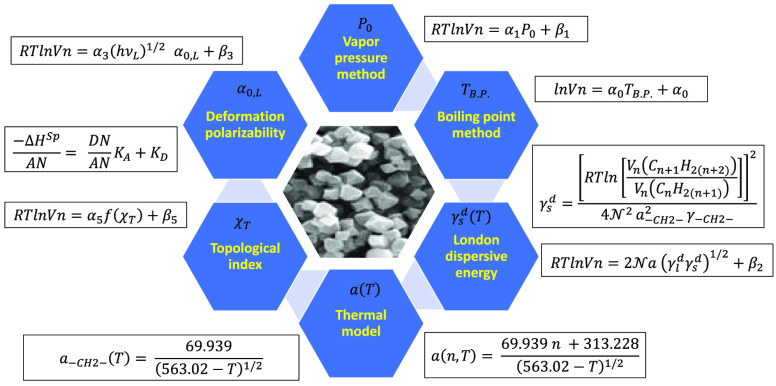

The London dispersive component of the surface energy of solid particles, their specific interactions, and Lewis acid–base parameters were usually calculated by using the inverse gas chromatography technique at infinite dilution. Serious irregularities were committed by some authors when using the Schultz method or Dorris–Gray relation. We proved that these methods cannot be used because they did not consider the important role of the temperature on variations in the surface area of solvents. We corrected in this paper some recent results by using new relations of the surface area of organic probes depending on the temperature and by using different molecular models using different geometries and mathematical calculations. The application of our new thermal model gave more precise results of the dispersive energy and Lewis’s acid–base constants of solid particles. The new thermal model was applied to several solid surfaces such as Ni-MOF-74, MgO, ZnO, TiO_2_, and Zn(OH)_2_. The obtained results showed a stronger Lewis basicity of MgO solid substrate and higher Lewis acidity of the Ni-MOF-74 surface.

## Introduction

Inverse gas chromatography (IGC) at infinite dilution can be considered a surface technique that gives interesting information on the surface thermodynamic properties of materials and nanomaterials, such as the dispersive surface energy, the specific free energy of adsorption, and the Lewis acid–base constants.^1−5^ The determination of these surface properties is very important to understand the behavior of materials during chemical synthesis, process engineering, wettability, adsorption, diffusion, evaporation, food technology, biomaterials, and pharmaceuticals. The dispersive surface energy and the specific properties of many solid particles were characterized by using IGC, such as oxides, metals,^6−10^ ceramic materials, and other solid substrates and nanostructures.^11−19^ The Lewis acid base constants of solid surfaces^20−23^ can be also determined by IGC from the determination of the specific variables. The dispersive surface energy of solid particles calculated by Dorris–Gray^24^ from the Fowkes relation^25^ was correlated to the work of adhesion *W*_a_ and the free energy of adsorption Δ*G*_a_^0^ by relation 1:
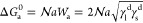
1Where *a* is the surface area of adsorbed molecule, γ_l_^d^ the dispersive component of the liquid solvent γ_l_^d^, and  Avogadro’s number. Dorris and Gray introduced the increment Δ*G*_–CH2–_^0^ of two consecutive *n*-alkanes C_*n*_H_2(*n*+1)_ and C_*n*_H_2(*n*+1)_:
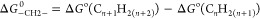
2They supposed that the surface area of the methylene group, *a*_–CH2–_, equal to 6 Å^2^ is constant. The surface energy γ_–CH2–_ of −CH_2_– is given by relation 3:
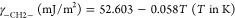
3Dorris and Gray^24^ proposed relation 4 giving the London dispersive surface energy of the solid γ_s_^d^:
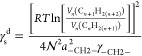
4Schultz et al.,^26^ using the Fowkes relation,^25^ proposed the expression of the free energy of adsorption Δ*G*_a_^0^:
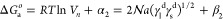
5Where γ_l_^d^and γ_s_^d^ are the respective dispersive components of the surface energy of the solvent and the solid, *a* is the surface area of adsorbed probes supposed constant, and the parameters α_2_ and β_2_ are constants of the problem. The determination of the retention volume of the various solvents allowed us to obtain both the γ_s_^d^ and Δ*G*_a_^sp^(*T*) of materials.

The Dorris–Gray and Schultz et al. methods used constant values of the surface areas of methylene group and organic probes. The use of Schultz et al. method^26^ to determine the specific variables of adsorption of polar molecules on the solid surfaces gave inaccurate results and consequently led to incorrect values of the Lewis acid–base parameters. Some new models and methods were recently proposed in literature^27−30^ to correct some irregularities on the surface properties and give more accurate results.

Many authors^7−9,11,13,14,31−34^ recently studied the London dispersive energy and the Lewis’s acid–base properties of some solid surfaces by using IGC technique and the classical methods of Dorris–Gray^24^ and Schultz et al.^26^ that were proved to give wrong results. Furthermore, in their study they used constant values of the surface areas of organic solvents and also supposed as constant the dispersive component of the surface tension of the various organic molecules.^7−9,31−34^

The results obtained by these authors^7−9,11,13,14,31−34^ cannot be considered as confident results, because of the following reasons:

(1) The dispersive component of the surface energy γ_l_^d^(*T*) of *n*-alkanes varied with the temperature. Table 1 gives the different equations γ_l_^d^(*T*) of the different nonpolar molecules.^27^ It was observed that many authors^31−34^ considered γ_l_^d^(*T*) as constant for the various *n*-alkanes.

**Table 1:** Equations of Dispersive Component of the Surface Energy γ_*l*_^*d*^ (mJ/m^2^) of *n*-Alkanes As a Function of the Temperature *T* (K)

*n*-alkanes	γ_l_^d^ (mJ/m^2^)
C_6_H_14_	γ_l_^d^ (C6) = −0.102*T* + 48.34
C_7_H_16_	γ_l_^d^ (C7) = −0.098*T* + 48.85
C_8_H_18_	γ_l_^d^ (C8) = −0.095*T* + 49.61
C_9_H_20_	γ_l_^d^ (C9) = −0.093*T* + 50.15

(2) Many scientists^7−9,11−14,31−34^ supposed *a*_–CH2–_ = 0.06 nm^2^. One proved in a previous study that *a*_–CH2–_(*T*) is function of the temperature. Hamieh^27^ proposed the expression of *a*_–CH2–_(*T*):
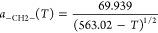
6And another general expression^27^ for *n*-alkanes
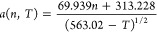
7and for polar solvents
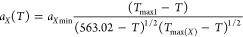
8Where *n* is the carbon atom number of *n*-alkane, *a*_–CH2–_(*T*), *a*(*n,T*), *a*_*X*_(*T*), and *a*_*X*min_ are the corresponding surface areas expressed in Å^2^ and *T*_Max__1_ and *T*_Max__(*X*)_ are two new intrinsic surface temperatures (in K) of polar molecules.^27^

(3) The surface areas of *n*-alkanes used in literature^7−14,31−34^ to determine the dispersive component of the surface energy of solid surfaces were that of Kiselev (Table 2). However, in previous papers,^35−37^ several ways were proposed to calculate the surface area of organic molecules by using different methods and models. Table 2 presents the different results.^27,35−37^

**Table 2:** Surface Areas of Various Molecules (in Å82) Given by the Various Models of van der Waals (VDW), Redlich–Kwong (R–K), Kiselev, Geometric, Cylindrical, or Spherical Models^27,35−37^

Molecules	VDW	Kiselev	Cylindrical	R-K	Spherical	Geometric
C_5_H_12_	47.0	45	39.3	36.8	36.4	32.9
C_6_H_14_	52.7	51.5	45.5	41.3	39.6	40.7
C_7_H_16_	59.2	57	51.8	46.4	42.7	48.5
C_8_H_18_	64.9	63	58.1	50.8	45.7	56.2
C_9_H_20_	69.6	69	64.4	54.5	48.7	64.0

Therefore, we must use these different values to give a comparison between the results obtained for the dispersive surface energy and the specific variables of adsorption of organic solvents on the solid surfaces.

In this paper, we were interested in the correction of the dispersive and specific surface properties of solid surfaces of some oxides and hydroxides such as Ni-MOF-74, MgO, ZnO, TiO_2_, and Zn(OH)_2_.

## Materials and Methods

One resumed in this section the different IGC methods used during the 40 past years by the scientific community. The oldest method was that proposed by Sawyer and Brookman^38^ that used the linearity between ln *V_n_* of adsorbed molecule, against the boiling point *T*_BP_ of the solvent probes:
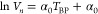
9with α_0_ and β_0_ constants depending on the solid-solvent interaction and experimentally determined by the parameters of the straight-line of *n*-alkanes.

The specific interactions of solids were determined by Saint-Flour and Papirer^39,40^ from the straight-line of *RT*ln *V_n_* = *f*(*P*_0_). where *P*_0_ is the vapor pressure of the solvent:
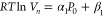
10where *R* is the ideal gas constant, *T* the absolute temperature, and α_1_ and β_1_ are constant parameters. The specific free energy of adsorption Δ*G*_a_^s*p*^ of solid substrates were determined from *RT*ln *V_n_* = *f*(*P*_0_) of a polar molecule, whereas the variations of Δ*G*_a_^sp^(*T*) against the temperature gave the values of the specific enthalpy Δ*H*_a_^s*p*^ and entropy Δ*S*_a_^sp^ of polar molecules. These specific variables allowed us to calculate the Lewis acid–base constants.

Several other IGC methods were proposed, and to characterize the solid surfaces, a similar linearity to separate the two dispersive and polar components of the specific interactions was found. The problem was the determination of the surface area of the organic molecules used in the IGC technique. As we previously evocated, there are many molecular models used to quantify the specific free energy of adsorption by applying the Fowkes relation and relation 4. This relation and the Dorris–Gray expression were used to obtain the value of γ_s_^d^ by supposing the surface areas of molecules as invariable parameter. Several molecular models based on the geometry of molecules, were used to determine the surface physicochemical properties of solid materials.^35−37^

The concept of the deformation polarizability α_0_ was proposed by Donnet et al.^41^ to characterize the specific interactions between solids and the adsorbed molecules (relation 11).
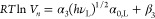
11where *ν*_L_ is the electronic frequency of the probe, *h* the Planck’s constant, and α_3_ and β_3_ constant parameters of interaction.

The method proposed by Chehimi et al.^42^ utilized the notion of the standard enthalpy of vaporization Δ*H*_vap_^0^ of solvents by supposing this parameter as constant:
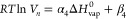
12Where α_4_ and β_4_ are two constants determined by the experimental values of *RT*ln *V_n_* and Δ*H*_vap_^0^. The Chehimi et al. method resembles the methods of Saint-Flour and Papirer (parameter ln *P*_0_) and Sawyer and Brookman (with *T*_BP_ as the parameter).

Brendlé and Papirer^43,44^ proposed the new parameter of topological index *X*_T_ that is related to the topology and the local electronic density in the polar probe structure (relation 13).
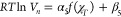
13Where α_5_ and β_5_ are two constants of adsorption.

In the different IGC methods and models, the experimental determination of Δ*G*_a_^s*p*^(*T*) of polar probes allowed us to obtain the specific enthalpy (−Δ*H*_a_^sp^) and entropy (Δ*S*_a_^sp^) of adsorbed molecules. The determination of Δ*H*_a_^s*p*^ of polar molecules led to evaluate the Lewis acid base constants *K*_A_ and *K*_D_ of solids by using relation 14 first used by Papirer et al.:^37,39,40^
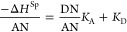
14AN and DN respectively representing the electron donor and acceptor numbers of the polar molecule were determined by Gutmann^45^ and corrected by Fowkes.^25^

On the other hand, one proposed, in previous studies,^28−30^ new entropic Lewis’s acid *ω*_A_ and base *ω*_D_ parameters (by analogy to the enthalpic Lewis’s acid *K*_A_ and base *K*_D_ parameters of a solid) determined by relations 15 or 16:
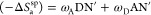
15or
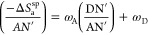
16In several studies,^27−30^ one proved the inaccuracy of the methods of Dorris–Gray and Schultz et al.,^26^ by showing the dependency of the surface area of molecules on the temperature.^27−30^ Consequently, the values of γ_*s*_^*d*^ obtained by several authors^7−14,31−34^ are not accurate and they have to be corrected.

The surface thermodynamic properties of some solid surfaces were corrected by using our new approach using the new expressions *a*(*T*) of the surface areas. We also used the classical IGC methods and models in order to show the large disparity between the obtained values of γ_s_^d^, Δ*G*_a_^sp^, and the specific properties of the studied materials.

The organic molecules (*n*-alkanes: pentane, hexane, heptane, octane, nonane; and polar molecules: acetonitrile, acetone; ethyl acetate, tetrahydrofuran, chloroform, dichloromethane, and nitromethane) and the solid particles (Ni-MOF-74, MgO, TiO_2_, ZnO, and Zn(OH)_2_) used in this paper were purchased from Aldrich. The solid particles of very high purity exhibited the following specific surface areas: Ni-MOF-74, 726.6 m^2^/g; MgO, 2 m^2^/g; TiO_2_, 59 m^2^/g; ZnO, 4 m^2^/g; and Zn(OH)_2_, 1.7 m^2^/g. The acceptor AN number of molecules was corrected by Riddle and Fowkes^46^ by giving a new acceptor number AN′ = AN – AN^*d*^, with AN*^d^* is relative to van der Waals dispersion forces. The acceptor and donor numbers were normalized by Hamieh et al.^35−37,47,48^ to become dimensionless parameters AN*′* and DN*′*.

The measurements of retention time of the various solvents were carried out with a Focus GC Chromatograph equipped with a flame ionization detector with high sensitivity. Stainless-steel columns were used with a 2 mm inner diameter and 20 cm length packed with 0.25 g of Ni-MOF-74, 1.5 g of titania particles, and 3.5 g for the other materials MgO, ZnO, and Zn(OH)_2_. The gas flow rate was set at a rate of 20 mL/min. The temperature of the injector and detector was maintained at 400 K during the experiments. A 1 μL Hamilton syringe was used to realize the injection of 0.1 μL of each probe vapor to approach the infinite dilution. The chromatographic columns were heated at temperature varying from 300 to 460 K. The retention time, *t*_R_, was determined by repeating three times the different injections of probes and limited the relative error to 1% in all IGC measurements.

## Results and Discussion

### Study of the Surface Properties of Ni-MOF-74

#### Study of the Dispersive Component of the Surface Energy of Ni-MOF-74

The dispersive components of the surface energy of Ni-MOF-74 surfaces were determined using the Dorris–Gray method,^24^ the molecular models, and the thermal model.^27−30,35−37^

**Figure 1:**
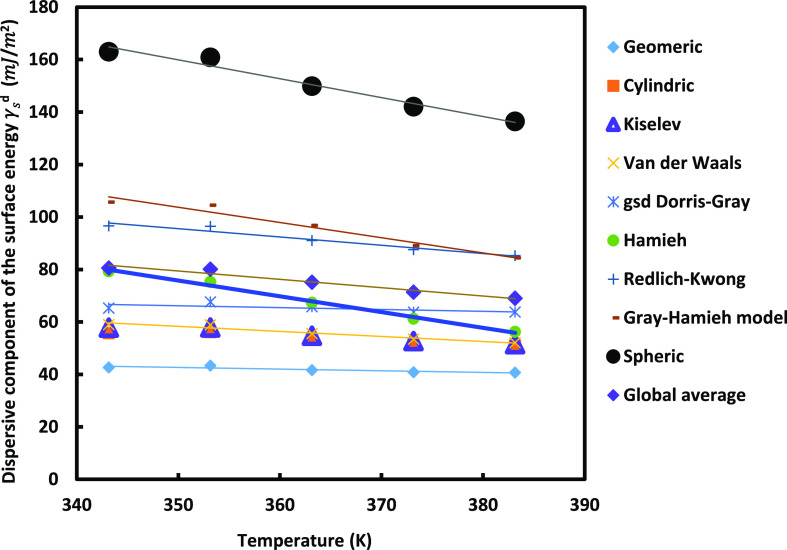
Evolution of γ_s_^d^ (mJ/m^2^) of Ni-MOF-74 as a function of temperature *T* (K).

We plotted, on Figure 1, γ_s_^d^(*T*) of Ni-MOF-74 solid particles against the temperature for all models. One observed that the methods and models used satisfied linear variations of γ_s_^d^(*T*) of the studied materials.

However, our results proved large deviations between the obtained values of γ_s_^d^(*T*). Indeed, there is an important influence of the IGC methods or models on the calculation of γ_s_^d^(*T*). For example, it was proved that the value of γ_s_^d^ obtained by the spherical model is three times larger than that of the Kiselev method (see Figure 1 and Table 3). This gave an importance to Hamieh’s works^27,28,30^ based on the thermal model that allowed more accurate results.

**Table 3:** Values of London Dispersive Surface Energy γ_s_^d^(*T*) of Ni-MOF-74 Particles at Various Temperatures for Different Models and Methods

	γ_s_^d^ for given *T*				
Model	343.15 K	353.15 K	363.15 K	373.15 K	383.15 K
Geometric	42.6	43.3	41.7	40.8	40.7
Cylindric	55.7	56.1	53.4	51.8	51.0
Kiselev	57.8	57.9	54.7	52.7	51.5
van der Waals	59.0	58.9	55.6	53.4	52.1
Dorris–Gray	65.3	67.7	65.8	63.7	63.7
Hamieh	79.3	75.3	67.4	61.2	56.2
Redlich–Kwong	96.6	96.5	91.0	87.5	85.3
Gray–Hamieh model	105.7	104.5	96.8	89.1	84.4
Spheric	163.0	160.9	149.9	142.1	136.4
Global average	80.5	80.1	75.1	71.4	69.1

From Table 3, Gray–Hamieh and spheric models gave larger values of γ_s_^d^, whereas the geometric model gave the smallest values. Comparable values were obtained by using Kiselev, van der Waals, and Dorris–Gray models and the moderate values were obtained by the Hamieh thermal model.

The error committed by the other models relative to the thermal model exceeds 25% with respect of Hamieh model.^27−30^ From Table 3, by taking the examples of the Kiselev method (γ_s_^d^(343.15 K ) = 57.8 mJ/m^2^)) and Hamieh model (γ_s_^d^(343.15 K) = 79.3 mJ/m^2^), one obtained an error equal to . These results clearly showed that the various models that did not consider the change in the surface areas of molecules when the temperature varied cannot give accurate values.

In order to distinguish between the different models, the expressions of γ_s_^d^(*T*) of materials were given on Table 4 as well as the dispersive surface entropy ε_s_^d^, the extrapolated values γ_s_^d^(*T* = 0 K). The values of the maximum of temperature *T*_Max_ calculated by the following relation:^27^ were also given on Table 4.

**Table 4:** Equations γ_s_^d^ (*T*) of Ni-MOF-74 Particles for All Used Molecular Models of *n*-Alkanes, ε_s_^d^(*T*), γ_s_^d^(*T* = 0 K), and *T*_Max_

Molecular model	γ_s_^d^(*T*) (mJ/m^2^)	ε_s_^d^ = d*γ*_**s**_^d^/d*T* (mJ m^–2^ K^–1^)	γ_s_^d^(*T* = 0 K) (mJ/m^2^)	*T*_Max_	*R*^2^
Geometric	γ_s_^d^(*T*) = −0.063*T* + 64.7	–0.063	64.7	1028.2	0.774
Cylindric	γ_s_^d^(*T*) = −0.136*T* + 102.8	–0.136	102.8	758.7	0.905
Kiselev	γ_s_^d^(*T*) = −0.178*T* + 119.5	–0.178	119.5	672.2	0.939
van der Waals	γ_s_^d^(*T*) = −0.192*T* + 125.5	–0.192	125.5	653.6	0.947
Dorris–Gray	γ_s_^d^(*T*) = −0.070*T* + 90.7	–0.070	90.7	1292.5	0.463
Hamieh	γ_s_^d^(*T*) = −0.601*T* + 286.2	–0.601	286.2	476.0	0.991
Redlich–Kwong	γ_s_^d^(*T*) = −0.316*T* + 206.1	–0.316	206.1	652.5	0.948
Gray–Hamieh	γ_s_^d^(*T*) = −0.580*T* + 306.7	–0.580	306.7	528.9	0.963
Spheric	γ_s_^d^(*T*) = −0.719*T* + 411.7	–0.719	411.7	572.3	0.971
Global average	γ_s_^d^(*T*) = −0.317*T* + 190.4	–0.317	190.4	600.3	0.958

Tables 3 and 4 showed that the results of the Redlich–Kwong model are closer to that of the thermal model showing the strong influence of the thermal agitation on the surface area and therefore on the dispersive surface energy of materials. The values of *T*_Max_ given on Table 4 are very different from one model to another. On obtained for Hamieh model *T*_Max_ ≈ 476*K* and larger values for the other models.

**Figure 2:**
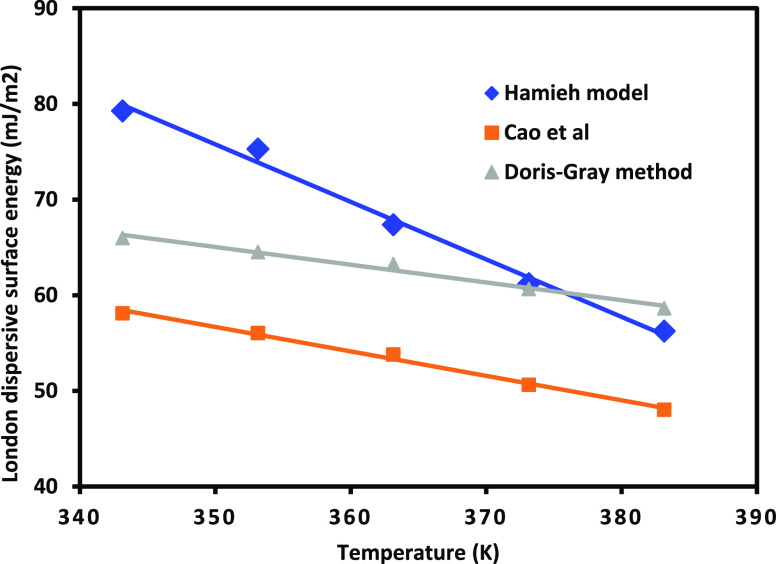
Evolution of γ_s_^d^(*T*) of Ni-MOF-74 particles against the temperature by comparing Dorris–Gray, Kiselev, and Hamieh models.

The methods of Dorris–Gray and Schultz et al. (using Kiselev results) were applied by Cao et al.^31^ and gave smallest values of γ_s_^d^ because these authors neglected the effect of the temperature. To compare between the results obtained by Cao et al.^31^ and that of the thermal model, we show in Figure 2 the curves of the dispersive component of the surface energy of solid particles as a function of the temperature. Figure 2 proves that there is a large difference between the classic models and that of the thermal model certainly due to the thermal effect on the surface area of organic molecules.

In the next section, we were interested in the determination of the specific surface properties of solid particles by comparing between the different models and methods and to prove that the specific properties of the material largely varied from one method to another.

#### Specific Enthalpy and Entropy, and Lewis Acid–Base Constants of Ni-MOF-74

**Figure 3:**
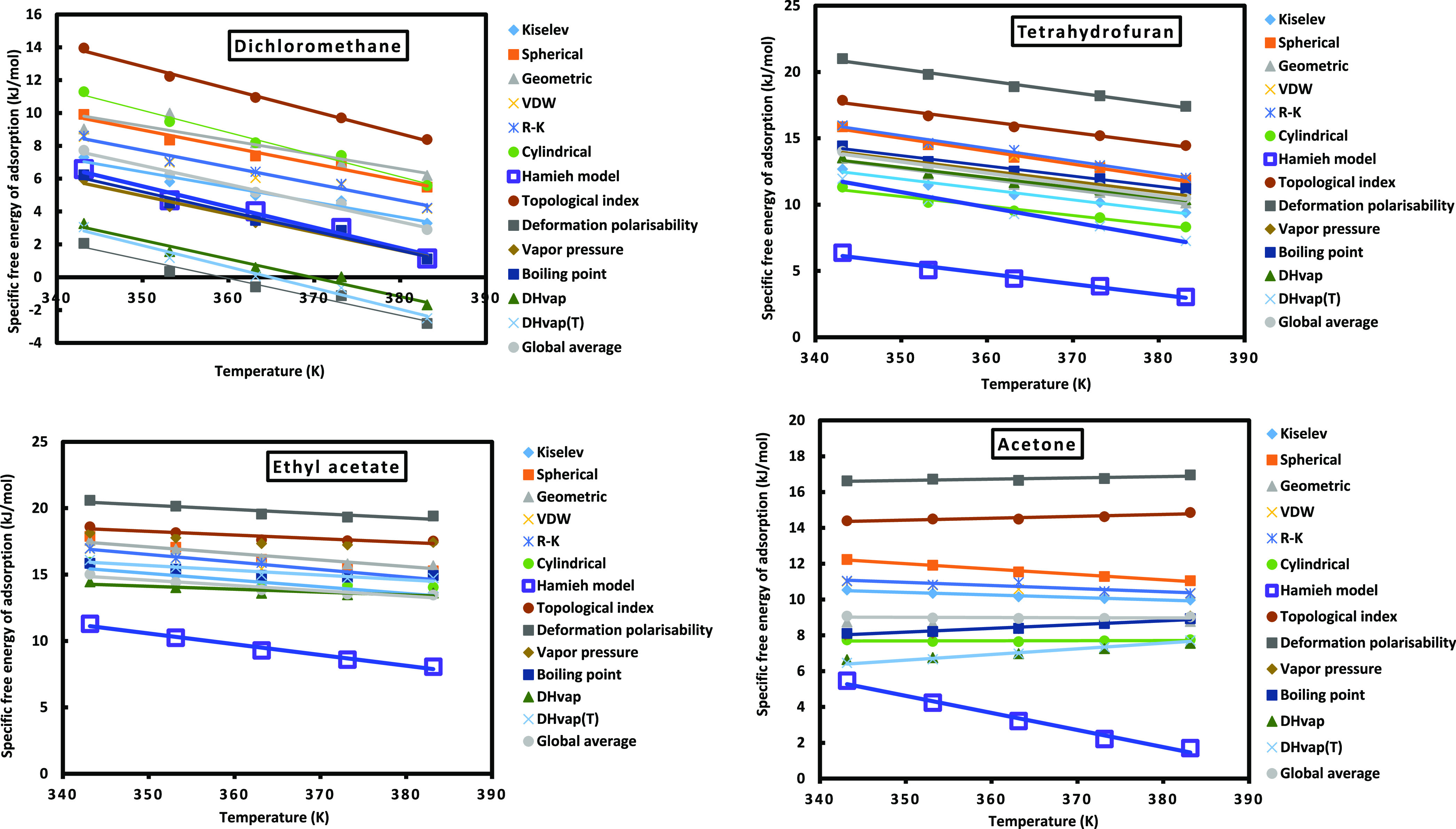
Variations of the Δ*G*_a_^sp^(*T*) of dichloromethane, THF, ethyl acetate, and acetone on Ni-MOF-74 solid particles for the different used methods.

Several molecular models were used to characterize Ni-MOF-74 solid particles, such as the thermal model with the vapor pressure,^39,40^ deformation polarizability,^41^ topological index, boiling point,^38^ and vaporization heat^42^ methods. The obtained results allowed calculation of the specific free energy (Δ*G*_a_^sp^ (*T*)) of polar molecules adsorbed on the solid particles (Figures 3 and Table S1). It was shown large disparities between the values of the specific free energy obtained by the various methods and models for the different polar solvents. The values of the specific free energy vary between 0 and 22 kJ/mol in absolute value. The highest values were obtained by the topological index and deformation polarizability methods. Some IGC methods such as that of the deformation polarizability and the enthalpy of vaporization gave negative values of Δ*G*_a_^*s*p^(*T*), meaning these methods are not valid in the domain of the specified temperature interval.

The values (−Δ*H*_a_^sp^) and (−Δ*S*_a_^sp^) respectively shown in Tables 5 and 6 proved important differences between the different methods and models.

**Table 5:** Variations of −Δ*H*_a_^sp^ (kJ mol^–1^) of the Polar Molecules Adsorbed on the Ni-MOF-74 Material for the Applied Methods

Model or method	CH_2_Cl_2_	THF	Ethyl acetate	Acetone
Geometric	55.14	41.03	34.21	8.36
Cylindric	57.30	35.70	32.40	7.46
Kiselev	38.54	39.44	32.70	15.33
van der Waals	42.96	48.43	36.17	16.88
Hamieh	41.70	37.19	38.91	0.14
Redlich–Kwong	43.25	48.65	36.35	17.06
Spheric	44.87	49.20	40.70	22.52
Topological index	–3.50	56.87	88.63	66.93
Deformation polarizability	40.38	51.02	31.46	14.17
Vapor pressure	43.84	41.82	24.67	–1.95
Boiling point	46.90	40.74	24.03	0.85
Δ*H*_vap_	42.27	39.36	21.85	–1.43
Δ*H*_vap_(*T*)	47.23	50.76	28.16	–4.47

**Table 6:** Variations of −Δ*S*_a_^sp^ (J K^–1^ mol^–1^) of the Polar Molecules Adsorbed on Ni-MOF-74 Material for the Applied Methods

Model or method	CH_2_Cl_2_	THF	Ethyl acetate	Acetone
Geometric	132	81	49	–1
Cylindric	135	72	49	–1
Kiselev	92	79	50	14
van der Waals	101	95	57	17
Hamieh	126	79	81	1
Redlich–Kwong	102	96	57	17
Spheric	103	98	67	30
Topological index	30	217	312	237
Deformation polarizability	112	88	32	–7
Vapor pressure	111	81	20	–29
Boiling point	119	77	24	–21
Δ*H*_vap_	114	76	22	–23
Δ*H*_vap_(*T*)	129	114	36	–32

On Tables 5 and 6, the large difference between the values of the specific enthalpy and specific entropy (−Δ*S*_a_^sp^ of dichloromethane, tetrahydrofuran (THF), ethyl acetate, and acetone adsorbed on Ni-MOF-74. Only the thermal model gave more accurate results because it considered the large effect of the temperature on the surface area. However, one observed that the different models gave values of the specific enthalpy (−Δ*H*_a_^sp^ of adsorption of dichloromethane comprised between 38.54 and 57.30 kJ mol^–1^ except for the topological index method that gave −3.50 kJ mol^–1^. In the case of the adsorption of THF, the values of (−Δ*H*_a_^sp^ were between 35.70 and 56.87 kJ mol^–1^, whereas for ethyl acetate, (−Δ*H*_a_^sp^ was found to be between 21.85 and 88.63 kJ mol^–1^. The adsorption of acetone gave different values (−Δ*H*_a_^sp^ varying from −4.47 to 66.93 kJ mol^–1^. It was also observed that an important effect of the method used on the values of the specific entropy of adsorption.

**Figure 4:**
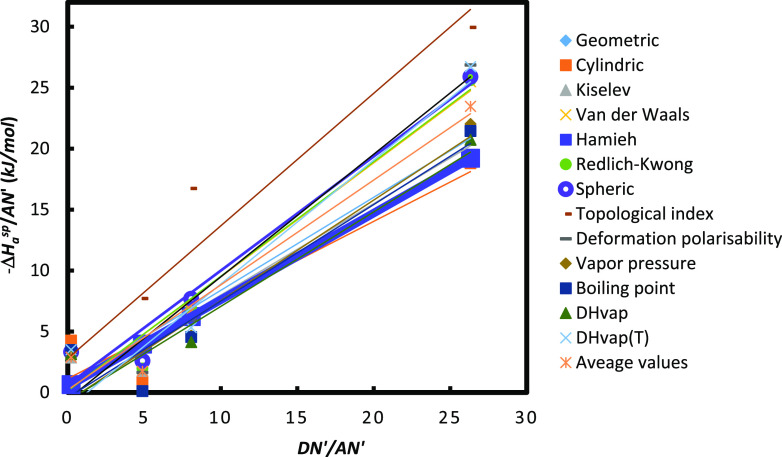
Variations of as a function of  of molecules adsorbed on Ni-MOF-74 particles.

The variations of  and  as a function of  of Ni-MOF-74 particles were represented on Figure 4 and Figure S1. For several methods, a good linearity was observed on the curves of Figure 4 and Figure S1, and this allowed us to obtain the Lewis acid–base constants of Ni-MOF-74. The slope of the curves on Figure 4 determined the value of their Lewis acid constant *K*_A_ and the ordinate at origin allowed to obtain their basic constant *K*_D_ for all tested models and methods. Figure 4 compares the different models and IGC methods by giving a global view of comparison. The greater the slope is, the greater the acidic constant is.

The values of the various Lewis’s enthalpic and entropic acid base constants *K*_A_, *K*_D_, *ω*_A_ and *ω*_D_ for all models and methods were determined and given by Table 7.

**Table 7:** Values of the Enthalpic Acid Base Constants *K*_A_ and *K*_D_ and the Entropic Acid Base Constants *ω*_A_ and *ω*_D_ of Ni-MOF-74 Particles with the Acid Base Ratios and Linear Regression Coefficients

Models and IGC methods	*K*_A_	*K*_D_	*K*_A_/*K*_D_	*R*^2^	10^–3^*ω*_A_	10^–3^*ω*_D_	*ω*_A_/*ω*_D_	*R*^2^
Geometric	0.46	0.44	1.04	0.908	0.90	0.30	2.95	0.8461
Cylindric	0.39	0.68	0.57	0.8856	0.78	0.82	0.94	0.8223
Kiselev	0.45	0.29	1.57	0.9526	0.90	–0.02	–40.67	0.9128
van der Waals	0.56	0.07	7.61	0.9528	1.10	–0.40	–2.77	0.9187
Hamieh	0.43	0.29	1.48	1.0000	0.86	1.39	0.62	1.0000
Redlich–Kwong	0.56	0.08	7.13	0.9529	1.11	–0.39	–2.83	0.9187
Spheric	0.56	0.37	1.51	0.9618	1.12	0.19	5.83	0.9355
Topological index	0.65	1.66	0.39	0.9209	2.47	5.92	0.42	0.9509
Deformation polarizability	0.60	–0.34	–1.75	0.9474	1.03	–1.21	–0.85	0.8568
Vapor pressure	0.49	–0.43	–1.13	0.9005	0.97	–1.86	–0.52	0.8128
Boiling point	0.47	–0.26	–1.81	0.8973	0.90	–1.13	–0.79	0.8072
Δ*H*_vap_	0.46	–0.42	–1.10	0.8971	0.88	–1.29	–0.69	0.8057
Δ*H*_vap_(*T*)	0.61	–0.77	–0.79	0.9052	1.37	–2.69	–0.51	0.851
Average values	0.51	0.13	4.08	-	1.11	–0.03	–38.69	-

The results of Table 7 clearly showed that the classical methods and models cannot be taken into consideration because they gave bad linear regression coefficient *R*^2^, with negative (inacceptable) values of acid–base parameters thus proving the nonvalidity of those models (deformation polarizability, vapor pressure, and enthalpy of vaporization methods). On the contrary, the thermal model gave more precise results with the highest linear regression coefficient *R*^2^ equal to 1.000 for the used solid material. It was shown that the Ni-MOF-74 surface is rather acidic than basic. At least four models gave similar results on the acidity of Ni-MOF-74 solid particles, but not the same values of the acid–base constants.

The use of the thermal model gave the results in Table 8.

**Table 8:** Values of the Enthalpic Acid Base Constants *K*_A_ and *K*_*D*_ and the Entropic Acid Base Constants *ω*_*A*_ and *ω*_*D*_ of Ni-MOF-74 Particles Using the New Thermal Model

Solid surface	*K*_A_	*K*_D_	*K*_A_/*K*_D_	10^–3^*ω*_A_	10^–3^*ω*_D_	*ω*_A_/ω_D_
**N**i-MOF-74	0.43	0.29	1.48	0.86	1.39	0.62

Our results proved the more acidic character of the Ni-MOF-74 surface *K*_A_*/K*_D_ = 1.48. The result obtained by Cao et al.^31^ also proved an acidic character for Ni-MOF-74 but with a ratio *K*_A_*/K*_D_ = 1.80 with a deviation of 22% with respect of the value obtained by our model. Even if one observed that the acid–base parameters are very close, however, the way with which Cao et al.^31^ proceeded to the calculation of the specific parameters was based on wrong values of the surface area of organic molecules. The specific free energy (−Δ*G*_a_^sp^ and enthalpy (−Δ*H*_a_^sp^ of adsorption of polar solvents on Ni-MOF-74 solid particles obtained by Cao et al. were largely different from those of the thermal model (Table 9).

**Table 9:** Values (kJ/mol) of the Specific Free Energy (−Δ*G*_a_^sp^(*T*)) and (−Δ*H*_a_^*s*p^) of the Various Polar Solvents Adsorbed on Ni-MOF-74 Particles Obtained by Cao et al.^31^ and the Hamieh Model^27^

*T* (K)	CH_2_Cl_2_	THF	Ethyl acetate	Acetone
–Δ*G*_a_^sp^(*T*)) of polar solvents usingthe Hamieh model^27^				
343.15	6.579	6.375	11.292	0.463
353.15	4.691	5.068	10.249	0.459
363.15	4.019	4.423	9.297	0.455
373.15	3.003	3.853	8.595	0.451
383.15	1.146	3.040	8.069	0.447
–Δ*H*_a_^sp^	41.70	37.19	38.91	0.45
–Δ*G*_a_^sp^(*T*) of polar solvents obtained by Cao et al^31^				
343.15	7.3	12.680	10.530	15.600
353.15	5.81	11.470	10.340	14.910
363.15	4.98	10.750	10.150	14.200
373.15	4.65	10.170	10.050	13.800
383.15	3.29	9.400	9.970	13.640
–Δ*H*_a_^sp^	38.7	39.660	15.370	32.950

Table 9 clearly showed large differences between the specific variables of Cao et al.^31^ and those obtained by applying the thermal model. This difference is due to the invalid Schultz method^26^ that considered the surface area of organic molecules as constant.^27−30^ Therefore, the larger influence of the thermal agitation on the surface area of chromatographic solvents has to be considered for more accurate results of the London dispersive energy and Lewis acid–base properties of solid surfaces.

### Study of the Surface Properties of MgO Particles

**Figure 5:**
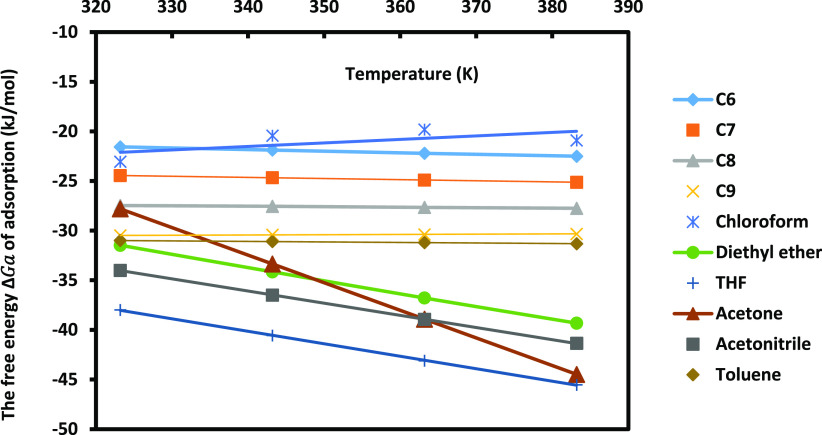
Evolution of the free energy Δ*G*_a_(*T*) of the various organic molecules adsorbed on MgO particles.

In order to extend the new thermal model, we applied the new findings to determine the surface properties of the magnesium oxide particles. The IGC technique led us to determine the retention volume of the different *n*-alkanes (from C6 to C9) and polar solvents (such as chloroform, diethyl ether, tetrahydrofuran, acetone, acetonitrile, and toluene) adsorbed on MgO surfaces. The determination of the retention volume of the adsorbed probes allowed to calculate the free energy of adsorption Δ*G*_a_(*T*) of the different organic solvents on MgO solid particles (Figure 5, Table S2).

**Figure 6:**
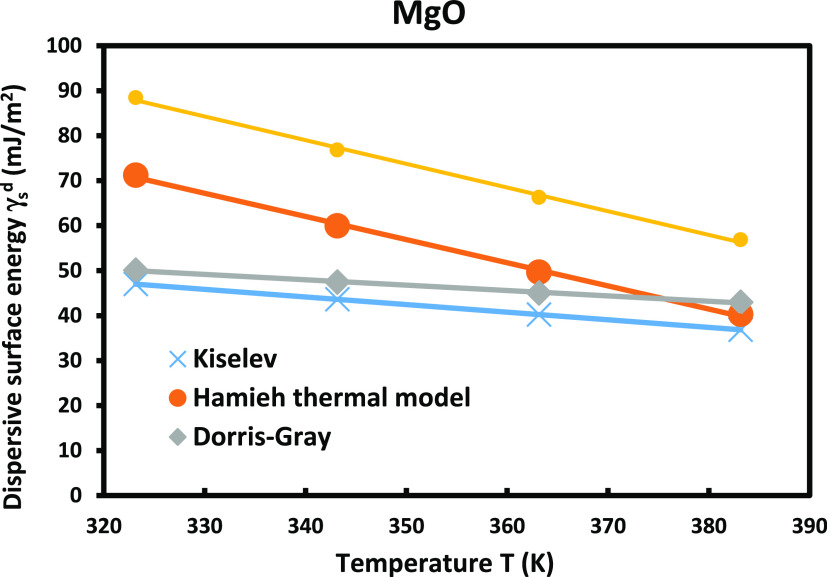
Variations of the dispersive surface energy γ_s_^d^(*T*) of the MgO surface for four molecular models.

Figure 5 showed linear variations of Δ*G*_a_(*T*) for the different organic solvents. The values of Δ*G*_a_(*T*) of *n*-alkanes adsorbed on MgO (Table S2) allowed by using the classical relation of Fowkes to draw on Figure 6 the variations of the London dispersive surface energy γ_s_^d^ (*T*) of MgO by using Kiselev results, Hamieh model, and Dorris–Gray and D–G–Hamieh models. Figure 6 shows different values of γ_s_^d^(*T*) obtained by the classical methods and that of the thermal model, with a deviation reaching more than 30% compared to the results of the Hamieh model.

The comparison between the values of the London dispersive surface energy of MgO particles for all models was given in Figure S2. It was shown that the spherical model overestimated the values of γ_s_^d^ of solid particles.

**Figure 7:**
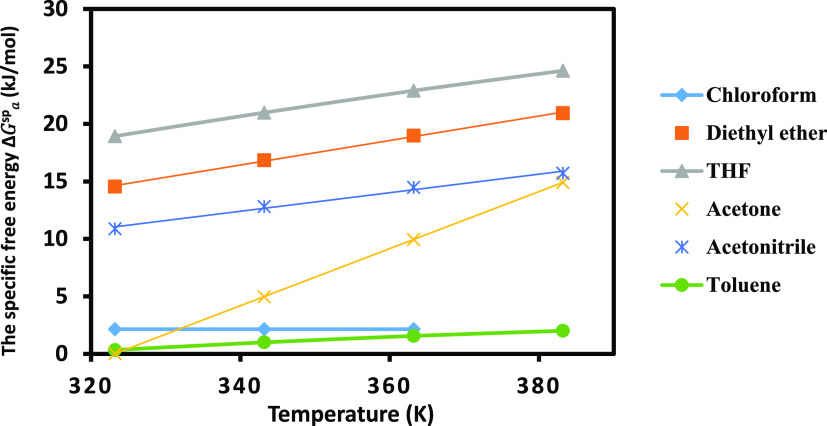
Evolution of the specific free energy (−Δ*G*_a_^sp^(*T*)) of the various organic molecules adsorbed on MgO particles using the Hamieh model.

By using the results of the free energy of adsorption of the different solvents (Table S2) and relation (5), we calculated the specific free energy of the different polar molecules. Figure 7 gave the curves of (−Δ*G*_a_^sp^(*T*)) of the polar solvents by using the thermal model (the other results were presented on Table S3). Figure 7 proved the amphoteric behavior of the MgO surface by showing the larger interaction energy with THF solvent for all temperatures. One found that this result was conformed to the larger ratio of donor and acceptor number of electrons of THF (.

**Figure 8:**
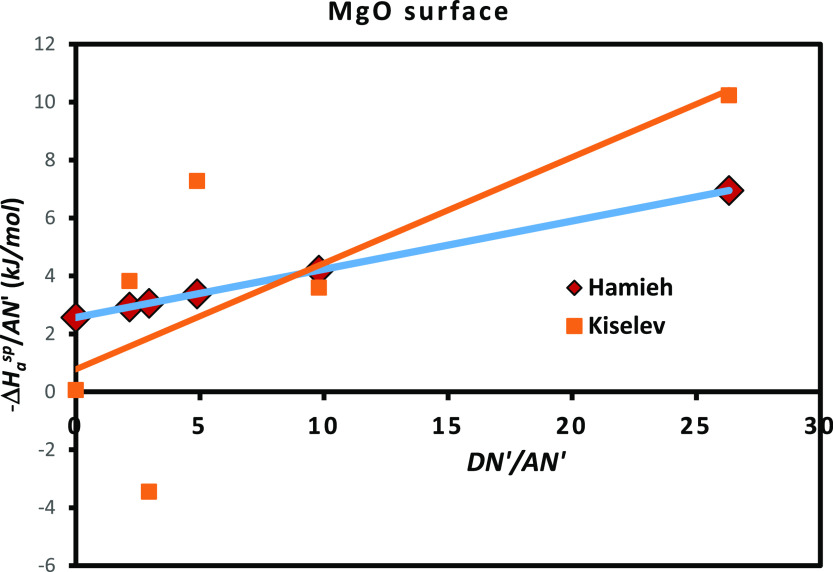
Variations of as a function of  of different polar molecules adsorbed on MgO for Hamieh model and the classical method.

In order to quantify the Lewis’s acid–base character of MgO, one used the results given by Table S3 and Figure 8 to draw the curves of Figures 8 and Figure S3 representing the variations of  and as a function of  of various solvents adsorbed on MgO.

It was shown in Figure 8 an excellent linearity obtained by the thermal Hamieh model relative to the poor linear correlation of the Kiselev method. The Lewis’s acid–base constants are presented in Table 10.

**Table 10:** Values of the Enthalpic Acid Base Constants *K*_A_ and *K*_D_ and the Entropic Acid Base Constants *ω*_A_ and *ω*_D_ of MgO Particles Using the Thermal Model and the Kiselev–Schultz Method

MgO	*K*_A_	*K*_D_	*K*_D_/*K*_A_	10^3^· *ω*_A_	10^3^· *ω*_D_	*ω*_D_/*ω*_A_
Hamieh model	0.067	1.026	15.39	0.70	2.17	3.09
Kiselev–Schultz	0.146	0.311	2.12	0.85	0.76	0.90

The results presented in Table 10 clearly showed the strong Lewis basicity of magnesium oxide particles. One observed a ratio of Lewis’s base/acid equal to 15.39 by using Hamieh model, whereas this ratio is about 2.12 by using the classical method.

### Comparison between the Surface Thermodynamic Properties of the Solid Particles

**Figure 9:**
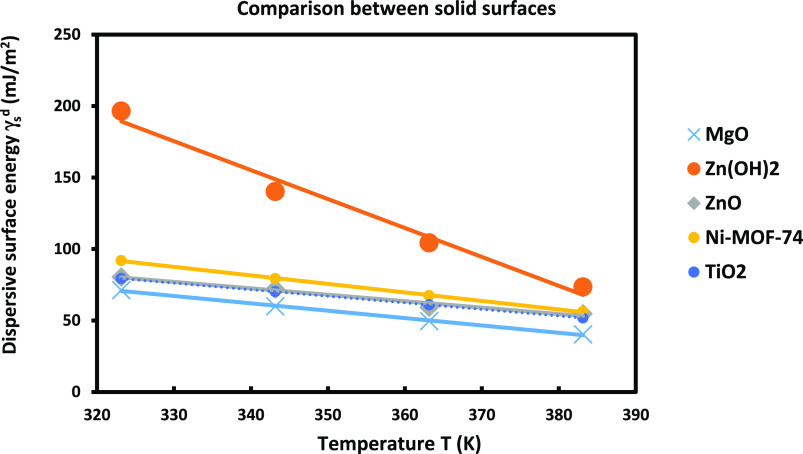
Variations of the dispersive surface energy γ_s_^d^(*T*) of different solid surfaces using the thermal model.

In this section, one presents the results obtained with the different solid substrates by using the thermal model. The results concerning the retention volumes obtained from the IGC measurements on the solid substrates ZnO, Zn(OH)_2_, and TiO_2_ were derived from previous results that were recently published.^49^ But the application of our new thermal models led to new results of the London dispersive energy given on Figure 9.

**Figure 10:**
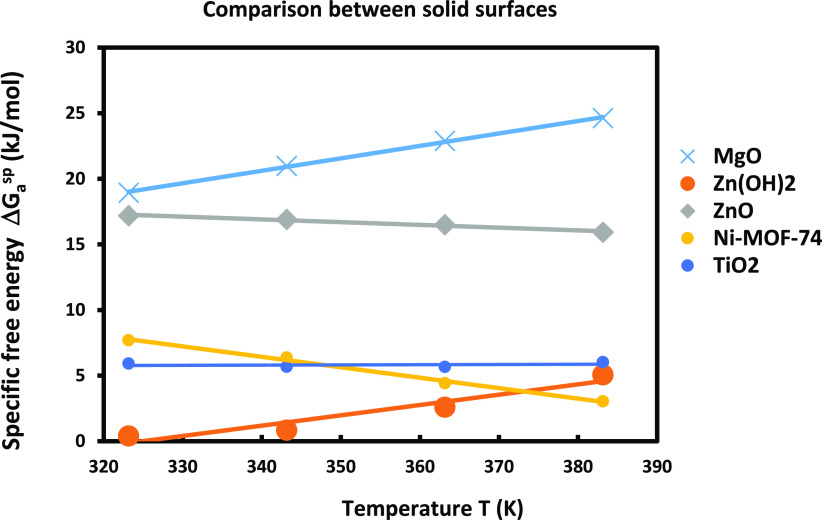
Evolution of −Δ*G*_a_^sp^(*T*)) of THF solvent adsorbed on the various solid particles using the Hamieh model.

The comparison between γ_s_^d^ (*T*) of the different solid materials was showed on Figure 9. One found that the obtained London dispersive surface energy of materials by using the Hamieh model allowed classification of the various solid surfaces in decreasing order of γ_s_^d^(*T*) (Figure 9):
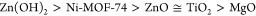
To compare the behavior of the different solid surfaces, one represented in Figure 10 the evolution of the specific surface energy of THF solvent adsorbed on the different materials drawn. The curves of Figure 10 once again proved that the higher basic surface is that of MgO that gave the stronger specific free energy followed by the ZnO surface.

To more understand the polar behavior of the various solid particles, one calculated the polar surface free energy (−Δ*G*_a_^sp^(*T*)_*S*_) of the adsorption of tetrahydrofuran on the previous materials by using the following relation:
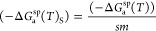
Where *s* is the specific surface area and *m* is the mass of the solid materials.

**Figure 11:**
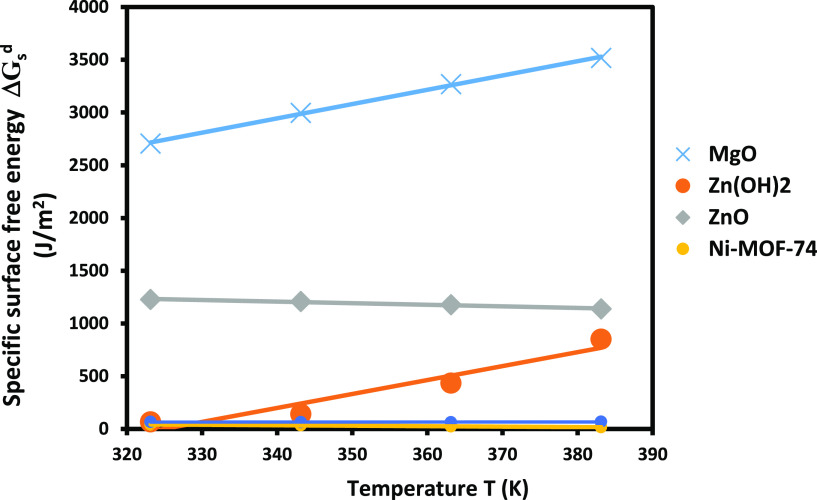
Evolution of the specific surface free energy −Δ*G*_a_^sp^(*T*)_S_) of THF solvent adsorbed on the various solid particles using the thermal model.

The obtained results allowed us to plot in Figure 11 the curves of the specific surface free energy (−Δ*G*_a_^sp^(*T*)_*S*_) (in J/m^2^) of THF adsorbed on the different materials. The comparison between Figures 10 and 11 led us to conclude that MgO gave the stronger polar interaction followed by ZnO and Zn(OH)_2_, whereas TiO_2_ and Ni-MOF-74 exhibited the least interaction with polar solvents.

To achieve the comparison between the Lewis’s acid–base properties of the various solid surfaces, one gave, in Table 11 the Lewis’s enthalpic and entropic acid–base constants of the solid surfaces.

**Table 11:** Values of the Enthalpic Lewis Acid Base Constants *K*_A_ and *K*_D_ and the Entropic Lewis Acid Base Constants *ω*_A_ and *ω*_D_ of the Various Solid Particles by Using the New Thermal Model

Solid surface	*K*_A_	*K*_D_	*K*_D_/*K*_A_	10^–3^*ω*_A_	10^–3^*ω*_D_	*ω*_D_/*ω*_A_
MgO	0.067	1.026	15.31	0.70	2.17	3.10
TiO2	0.067	0.651	9.76	0.04	3.33	87.00
Zn(OH)2	0.234	0.372	1.59	0.69	0.76	1.10
Ni-MOF-74	0.430	0.290	0.67	0.86	1.39	1.62
ZnO	0.184	0.095	0.51	0.14	0.82	5.90

Table 11 led to following comparison of the Lewis basicity of the solid materials:
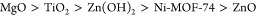
One deduced that the MgO surface exhibited the highest Lewis’s basicity followed by the TiO_2_ surface. This result was conformed by the inverse tendency of the London dispersive surface energy, giving the lowest value of γ_s_^d^ of MgO solid particles. On the other hand, the highest Lewis basicity was also confirmed in other studies by measuring the zeta potential of MgO particles dispersed in the different polar organic media and in aqueous medium.^47,48^

## Conclusion

Several molecular models and IGC methods were applied to characterize the surface properties of different solid particles such as Ni-MOF-74, MgO, ZnO, TiO_2_, and Zn(OH)_2_. The dispersive component of the surface energy of Ni-MOF-74 was obtained by applying various molecular models. The best results were obtained by the Hamieh model that corrected the values of the surface areas of molecules for the different values of the temperature. We proved the nonvalidity of the Schultz method. A correction was performed using the thermal model and compared with the other models and methods.

The determination of the surface free energy −Δ*G*_a_^0^(*T*) of adsorption and the specific free enthalpy Δ*G*_a_^sp^(*T*), gave the values of the specific parameters such as Δ*H*_a_^sp^ and *S*_a_^sp^ of the different polar solvents adsorbed on Ni-MOF-74 by using various molecular models and chromatographic methods. The evaluation of the surface properties of MgO particles proved lower dispersive surface energy but stronger Lewis’s surface basicity with a ratio of Lewis’s base/acid equal to 15.39 by using Hamieh model. The comparison between the different surfaces proved the highest basicity of MgO followed by the titanium dioxide.

## Supporting Information Available

Table S1, values of the specific free energy (−-Δ*G*_a_^sp^(*T*)) of the various polar solvents adsorbed on Ni-MOF-74 particles at different temperatures using the various molecular models and IGC method; Table S2, values of the free energy (−Δ*G*_a_(*T*)) of the various organic molecules adsorbed on MgO particles at different temperatures; Table S3, values of the specific free energy (−Δ*G*_a_^sp^(*T*)) of the various polar solvents adsorbed on MgO particles at different temperatures by using the Hamieh model and the Kiselev method; Figure S1, variations of  as a function of  for different molecular models and IGC methods for Ni-MOF-74 particles; Figure S2, variations of the dispersive surface energy γ_s_^d^ (*T*) of MgO surface for all molecular models; Figure S3, variations of  as a function of  of different polar molecules adsorbed on MgO particles for the Hamieh model and the Kiselev method.(PDF)
