# Supplementary material for: Some Irregularities in the Evaluation of Surface Parameters of Solid Materials by Inverse Gas Chromatography
Source: Langmuir. 2023 Nov 21;39(48):17059–70. doi: 10.1021/acs.langmuir.3c01649 (PMC10702619; doi:10.1021/acs.langmuir.3c01649)
Supplement: Supplementary file 1 — la3c01649_si_001.pdf [file la3c01649_si_001.pdf]

Supporting information

Some Irregularities in the Evaluation of Surface Parameters of Solid Materials by Inverse Gas Chromatography

Tayssir Hamieh[1,2]

[1]Faculty of Science and Engineering, Maastricht University, P.O. Box 616, 6200 MD Maastricht, Netherlands
[2]Laboratory of Materials, Catalysis, Environment and Analytical Methods Laboratory (MCEMA), Faculty of Sciences, Lebanese University, Hadath, Lebanon

Correspondence: Faculty of Science and Engineering, Maastricht University, P.O. Box 616, 6200 MD Maastricht, The Netherlands, E-mail: t.hamieh@maastrichtuniversity.nl

Table S1. Values (in kJ/mol) of the specific free energy ($-\Delta G_a^{sp}(T)$) of the various polar solvents adsorbed on Ni-MOF-74 particles at different temperatures by using the various molecular models and IGC methods.

ΔG_a^{sp} (T) (in kJ/mol)	Kiselev			
**T(K)**	**CH_2Cl_2**	**THF**	**Ethyl acetate**	**Acetone**
343.15	7.300	12.680	15.600	10.530
353.15	5.810	11.470	14.910	10.340
363.15	4.980	10.750	14.200	10.150
373.15	4.650	10.170	13.800	10.050
383.15	3.290	9.400	13.640	9.970

ΔG_a^{sp} (T) (in kJ/mol)	Spherical			
**T(K)**	**CH_2Cl_2**	**THF**	**Ethyl acetate**	**Acetone**
343.15	9.911	15.869	17.882	12.237
353.15	8.351	14.517	17.057	11.909
363.15	7.382	13.571	16.154	11.540
373.15	6.941	12.794	15.585	11.280
383.15	5.487	11.845	15.272	11.048

ΔG_a^{sp} (T) (in kJ/mol)	Geometric			
**T(K)**	**CH_2Cl_2**	**THF**	**Ethyl acetate**	**Acetone**
343.15	9.988	13.493	17.568	8.742
353.15	8.185	12.274	16.924	8.676
363.15	6.948	11.522	16.205	8.657
373.15	6.196	10.917	15.812	8.710

383.15	4.373	10.128	15.677	8.774

ΔG_a^{sp} (T) (in kJ/mol)		VDW		
**T(K)**	**CH_2Cl_2**	**THF**	**Ethyl acetate**	**Acetone**
343.15	8.545	15.925	16.944	11.010
353.15	6.984	14.598	16.211	10.793
363.15	6.051	13.675	15.425	10.567
373.15	5.630	12.924	14.962	10.439
383.15	4.171	11.997	14.745	10.326

ΔG_a^{sp} (T) (in kJ/mol)		R-K		
**T(K)**	**CH_2Cl_2**	**THF**	**Ethyl acetate**	**Acetone**
343.15	8.611	15.972	16.972	11.039
353.15	7.048	14.644	16.239	10.822
363.15	6.428	14.121	15.893	10.957
373.15	5.684	12.964	14.984	10.462
383.15	4.220	12.034	14.765	10.347

ΔG_a^{sp} (T) (in kJ/mol)		Cylindr.		
**T(K)**	**CH_2Cl_2**	**THF**	**Ethyl acetate**	**Acetone**
343.15	11.291	11.329	15.928	7.737
353.15	9.473	10.166	15.263	7.652
363.15	8.190	9.529	14.566	7.640
373.15	7.414	9.022	14.183	7.694
383.15	5.586	8.317	14.043	7.749

ΔG_a^{sp} (T) (in kJ/mol)		Hamieh model		
**T(K)**	**CH_2Cl_2**	**THF**	**Ethyl acetate**	**Acetone**
343.15	6.579	6.375	11.292	0.463
353.15	4.691	5.068	10.249	0.459
363.15	4.019	4.423	9.297	0.455
373.15	3.003	3.853	8.595	0.451
383.15	1.146	3.040	8.069	0.447

ΔG_a^{sp} (T) (in kJ/mol)		Topological index		
**T(K)**	**CH_2Cl_2**	**THF**	**Ethyl acetate**	**Acetone**
343.15	13.955	17.870	18.603	14.391
353.15	12.225	16.690	18.160	14.490
363.15	10.948	15.861	17.619	14.489
373.15	9.600	15.200	17.550	14.620
383.15	8.386	14.457	17.527	14.856

ΔG_a^{sp} (T) (in kJ/mol)	Deformation polarizability			
**T(K)**	**CH_2Cl_2**	**THF**	**Ethyl acetate**	**Acetone**
343.15	2.060	21.005	20.599	16.623
353.15	0.350	19.819	20.152	16.716
363.15	-0.586	18.900	19.553	16.650
373.15	-1.125	18.210	19.327	16.753
383.15	-2.821	17.411	19.407	16.958

ΔG_a^{sp} (T) (in kJ/mol)	Vapor pressure			
**T(K)**	**CH_2Cl_2**	**THF**	**Ethyl acetate**	**Acetone**
**343.15**	5.933	14.153	18.130	7.935
**353.15**	4.296	12.933	17.779	8.118
**363.15**	3.325	12.167	17.337	8.375
**373.15**	2.789	11.559	17.239	8.710
**383.15**	1.132	10.779	17.424	9.070

ΔG_a^{sp} (T) (in kJ/mol)	Boiling point			
**T(K)**	**CH_2Cl_2**	**THF**	**Ethyl acetate**	**Acetone**
343.15	6.234	14.430	15.847	8.099
353.15	4.518	13.257	15.410	8.208
363.15	3.463	12.527	14.948	8.388
373.15	2.850	11.959	14.810	8.648
383.15	1.111	11.215	14.929	8.926

ΔG_a^{sp} (T) (in kJ/mol)	ΔHvap			
**T(K)**	**CH_2Cl_2**	**THF**	**Ethyl acetate**	**Acetone**
343.15	3.269	13.545	14.455	6.639
353.15	1.557	12.372	14.018	6.751
363.15	0.587	11.668	13.597	6.972
373.15	0.026	11.114	13.483	7.257
383.15	-1.682	10.383	13.619	7.552

ΔG_a^{sp} (T) (in kJ/mol)	ΔHvap(T)			
**T(K)**	**CH_2Cl_2**	**THF**	**Ethyl acetate**	**Acetone**
343.15	3.055	11.963	16.101	6.462
353.15	1.179	10.356	15.547	6.664
363.15	0.060	9.288	14.968	6.972
373.15	-0.645	8.372	14.720	7.340
383.15	-2.504	7.267	14.732	7.707

Table S2. Values (in kJ/mol) of the free energy ($-\Delta G_a(T)$) of the various organic molecules adsorbed on MgO particles at different temperatures.

Temperature (K)	323.2	343.2	363.2	383.2
**C6**	21.564	21.884	22.209	22.500
**C7**	24.452	24.673	24.908	25.126
**C8**	27.467	27.552	27.656	27.750
**C9**	30.490	30.423	30.380	30.330
**Chloroform**	23.048	20.431	19.816	20.907
**Diethyl ether**	31.479	34.152	36.789	39.319
**THF**	37.991	40.564	43.101	45.531
**acetone**	27.814	33.355	38.920	44.481
**Acetonitrile**	34.004	36.485	38.951	41.353
**Toluene**	31.001	31.092	31.207	31.319

Table S3. Values (in kJ/mol) of the specific free energy ($-\Delta G_a^{sp}(T)$) of the various polar solvents adsorbed on MgO particles at different temperatures by using hamieh model and Kiselev method.

**Kiselev method**				
**Temperature (K)**	323.15	343.15	363.15	383.15
**TCM**	3.504	2.230	3.720	
**Diethyl ether**	15.829	17.899	19.969	22.039
**THF**	15.466	17.688	19.863	21.950
**Acetone**	10.442	15.008	19.574	24.140
**Acetonitrile**	-0.853	2.926	6.727	10.562
**Toluene**	3.283	2.783	2.283	1.783
**Hamieh model**				
**Temperature (K)**	323.2	343.2	363.2	383.2
**Chloroform**	2.147	2.150	2.149	
**Diethyl ether**	14.543	16.839	18.985	20.933
**THF**	18.939	20.978	22.889	24.627
**Acetone**	0.027	4.973	9.951	14.906
**Acetonitrile**	10.881	12.805	14.477	15.709
**Toluene**	0.336	1.000	1.558	2.006

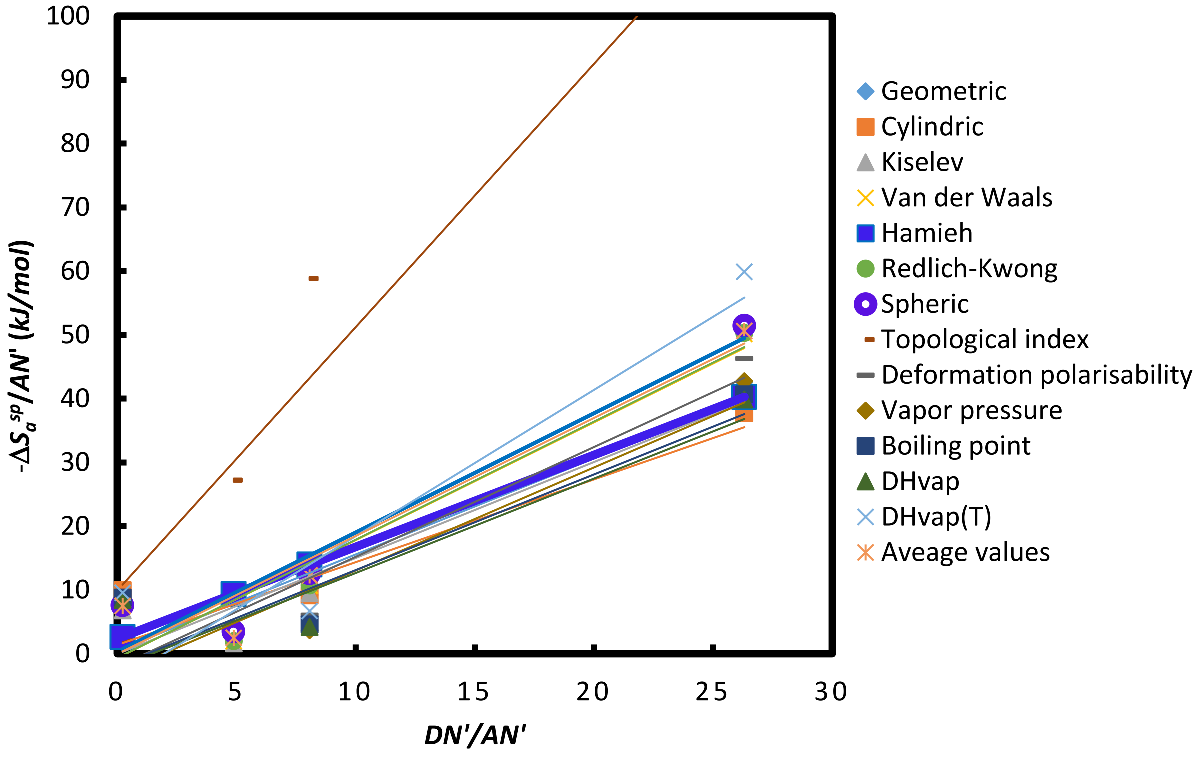

Figure S1. Variations of $\left(\frac{-\Delta S_a^{sp}}{AN'}\right)$ as a function of $\left(\frac{DN'}{AN'}\right)$ for different molecular models and IGC methods for Ni-MOF-74 particles.

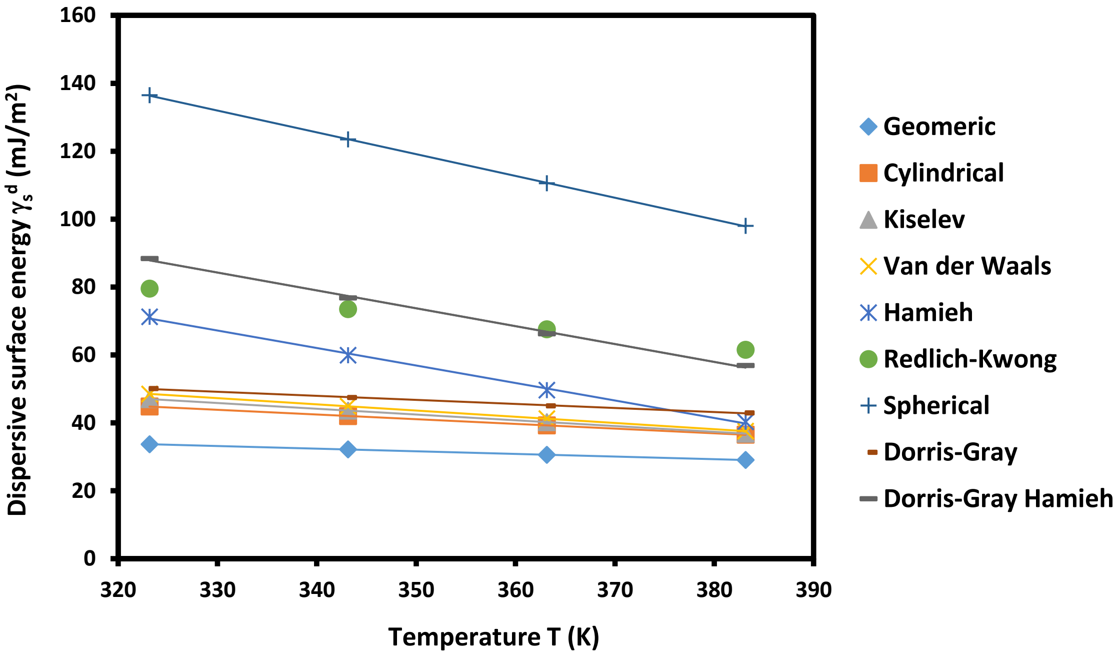

Figure S2. Variations of the dispersive surface energy $\gamma_s^d(T)$ of MgO surface for all molecular models.

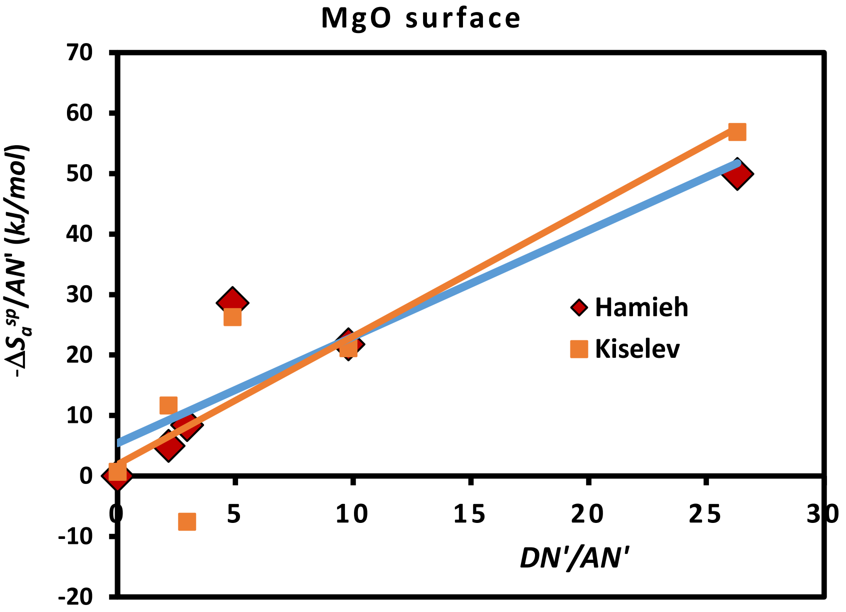

Figure S3. Variations of $\left(\frac{-\Delta S_a^{sp}}{AN'}\right)$ as a function of $\left(\frac{DN'}{AN'}\right)$ of different polar molecules adsorbed on MgO particles for Hamieh model and Kiselev method.
